# Integrating viscoelastic mass spring dampers into position-based dynamics to simulate soft tissue deformation in real time

**DOI:** 10.1098/rsos.171587

**Published:** 2018-02-14

**Authors:** Lang Xu, Yuhua Lu, Qian Liu

**Affiliations:** 1 Britton Chance Center for Biomedical Photonics, School of Engineering Sciences, Wuhan National Laboratory for Optoelectronics-Huazhong University of Science and Technology, Hubei, Wuhan 430074, People's Republic of China; 2 Key Laboratory for Biomedical Photonics, Huazhong University of Science and Technology, Ministry of Education, 1037 Luoyu Road, Hubei, Wuhan 430074, People's Republic of China

**Keywords:** soft tissue deformation, mass spring dampers, position-based dynamics, virtual surgery

## Abstract

We propose a novel method to simulate soft tissue deformation for virtual surgery applications. The method considers the mechanical properties of soft tissue, such as its viscoelasticity, nonlinearity and incompressibility; its speed, stability and accuracy also meet the requirements for a surgery simulator. Modifying the traditional equation for mass spring dampers (MSD) introduces nonlinearity and viscoelasticity into the calculation of elastic force. Then, the elastic force is used in the constraint projection step for naturally reducing constraint potential. The node position is enforced by the combined spring force and constraint conservative force through Newton's second law. We conduct a comparison study of conventional MSD and position-based dynamics for our new integrating method. Our approach enables stable, fast and large step simulation by freely controlling visual effects based on nonlinearity, viscoelasticity and incompressibility. We implement a laparoscopic cholecystectomy simulator to demonstrate the practicality of our method, in which liver and gallbladder deformation can be simulated in real time. Our method is an appropriate choice for the development of real-time virtual surgery applications.

## 1. Introduction

Surgery simulators can help novices become familiar with real surgical procedures without human loss. They enable scientific and repeatable training through visual rendering via software and interactive feedback via a hardware-based manipulator. Compared with actual surgical training, simulators reduce the cost of learning and guarantee the training's effectiveness [1]. Simulating the deformation of soft tissue is necessary for rendering three key aspects of a surgery simulator: the surgical environment, organs' interactions with the manipulator and force feedback [2]. A well-balanced deformation method can more closely simulate clinical manifestations. Further, accurate simulation of deformation, tearing, bleeding and force feedback make training more efficient. Modelling soft tissue as a viscoelastic body represents more complex nonlinear behaviours than a simple elastomer and viscous body, including geometric features such as creep, stress relaxation and incompressibility [3]. Therefore, it is crucial to develop a stable and fast method with which to simulate the complex deformation behaviours of biological soft tissue in real time.

The development of a real-time deformation algorithm has been accompanied by improvements in computing hardware. In early work, poor computational performance required the use of geometric techniques to simulate deformation, including spline and patch, freeform deformation and skin-based animation. In these techniques, physical accuracy is sacrificed for computational efficiency, and the system has no knowledge about the material being deformed [4]. With advances in computing power, two types of physics-based approaches have flourished. One solves partial differential equations based on a continuum model. This approach is typified by a finite-element method based on meshing object space, and uses the same strategy as the finite-volume and finite-differential methods [5]. The second approach, a meshless method based on shape functions and support domains has been used increasingly widely, such as with smoothed particle hydrodynamics and radial basis function methods [6]. The advantage of continuum methods is that they are sufficiently accurate to reflect an object's physical characteristics. They are widely used in structural analysis and electromagnetic radiation calculation [7]. Owing to deformation introducing the mesh distortion which reduced accuracy, finite-element method is naturally difficult to deal with large deformation. But the meshless method does not require connection information between simulation node, it works well under large deformation. Also, the material point method uses particles called material point to keep the physical properties and calculate the deformation gradient under background grid, it has been widely used in large deformation, multiphase simulation and fluid dynamics [8]. However, these methods are prohibitively time consuming, and are usually employed in offline applications. Our simulation aims to reflect the significant characteristics of soft tissue (such as viscoelasticity and incompressibility) in surgical applications; for real-time applications, it is appropriate to choose a physics-like method, using mass spring dampers (MSD) [9] and position-based dynamics (PBD) [10]. By abstracting typical features into this model, we achieve high computational efficiency.

Unlike the finite-element method, which uses the discrete solution domain of a partial difference equation, the mass spring method models discrete objects directly. An object consists of node masses with no size and elastic springs with no mass. Deformation is generated by applying elastic force to a node. The state of the system can then be determined by solving algebraic equations derived from Hooke's Law and Newton's second law. The mass spring method is simple to calculate and has a clearly structured topology; however, because nodes can only affect adjacent nodes, force propagation is limited and somewhat delayed, which causes local superelasticity [11]. A volume mesh topology and nonlinear spring can mitigate this effect [12]. When updating the node position, numerical integration is required for iteration. An explicit Euler step would cause the result to fail to converge, owing to second-order error. Using implicit Verlet or Runge–Kutta integration can improve solution accuracy [13]. Deformation in the model is directly related to spring stiffness, but the physical properties only indicate blurry correlation with spring stiffness. Therefore, it is difficult to adjust the parameters of MSD, which is a pressing concern for their application [14].

**Figure 1.**
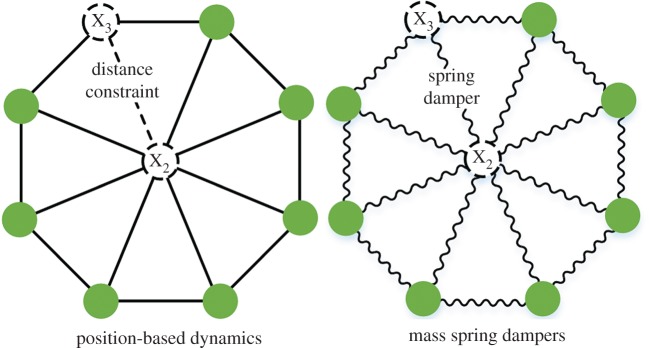
The same strain limit effects of separate description in PBD by distance constraint and in MSD by a spring damper.

As with MSD, PBD discretizes an object as a set of node masses (figure 1), although the relationship between nodes is described as a constraint function. The constraint function is a scalar function that takes node positions as variables. When the constraint equilibrium state changes, node position is modified via constraint projection iteration to satisfy the constraint function [15]. Therefore, deformation calculation is a constraint-function optimization problem. Different constraint functions enable the realization of multiple deformation features. For example, distance and dihedral angle constraints are used to simulate cloth [16]. Strain energy constraint is used to simulate soft bodies [17]. A Darboux frame is used to simulate an elastic rod [18]. Depending on the Gauss–Seidel method used to directly modify the node position, the PBD method is unconditionally stable. Despite its many advantages, the conventional PBD method has two notable problems. One is that constraint stiffness and deformation effects depend on iteration count and timestep, while the iteration count determines the constraint convergence and computation costs. Hence, it is necessary to decouple these parameters to more easily control the model. The second problem is that no force or time parameters participate in the constraint projection step. Thus, it is difficult to simulate the viscoelasticity and nonlinearity of soft tissue, as these are time-related force characteristics. The distance-dependent variable constraint parameter can be used to fit the nonlinearity by controlling the node position directly [19]. However, due to the lack of time and force parameters in constraint projection step, the viscoelasticity cannot be integrated into.

This study proposes a new method for integrating viscoelastic and nonlinear springs into PBD. It realizes fast and stable simulation of rich and complex deformation behaviour, such as the viscoelasticity, nonlinearity and incompressibility of biological soft tissue. In this method, deformation effects do not depend on the iteration count and timestep. The contributions of this study are as follows:

— a new method of integrating viscoelastic and nonlinear springs into PBD;
— examples that demonstrate the validity of the proposed method compared with analytical solutions of viscoelasticity;
— a simulation of liver and gallbladder deformation, demonstrating the different structures of the organs;
— a laparoscopic cholecystectomy simulator that demonstrates the practical applicability of the proposed method.

## 2. Material and methods

### 2.1. Integration method

The objects that we aim to simulate are each composed of a set of mass points, constraints and springs. The goal of deformation calculation is to find the actual position of every mass point at each step. The position of a mass point is controlled by its total force load. For our method, based on Newton's second law of motion

2.1 $${\text{f}}_{\mathrm{total}}={\text{f}}_{\mathrm{constraint}}+{\text{f}}_{\mathrm{spring}}+{\text{f}}_{\mathrm{external}}=\text{M}\ddot{\text{x}},$$

where $\text{x}={\left[\begin{array}{cccc} x_{1} & x_{2} & \ldots & x_{n} \end{array}\right]}^{T}$ is the column vector of the mass point position, the $\ddot{\text{x}}$ is the second-order derivative of mass point position to time which are commonly described as the acceleration of mass points. **M** is the diagonal matrix of point masses, ${\text{f}}_{\mathrm{constraint}}={\left[\begin{array}{cccc} f_{c1} & f_{c2} & \ldots & f_{cm} \end{array}\right]}^{T}$ is the column vector of constraint forces, and ${\text{f}}_{\mathrm{spring}}$ and ${\text{f}}_{\mathrm{external}}$ are the column vector of the spring and external forces, respectively.

To determine ${\text{f}}_{\mathrm{constraint}}$, constraint energy potential is defined as

2.2 $$\text{U}(\text{x})=\frac{1}{2}\text{C}{\left(\text{x}\right)}^{T}\alpha^{-1}\text{C}(\text{x}),$$

where $\text{C}(\text{x})={\left[\begin{array}{cccc} C_{1} & C_{2} & \ldots & C_{m} \end{array}\right]}^{T}$ is a column vector of the constraint function, and **α** is a block diagonal compliance matrix corresponding to the inverse stiffness of the constraint. As for the elastic and gravity potential, the force generated by the energy potential is in the direction of the maximum energy change to the mass point position. Thus, the constraint force is defined as the gradient of the mass point position of energy potential:

2.3 $${\text{f}}_{\mathrm{constraint}}=-\nabla_{\text{X}}{\text{U}}^{T}=-\nabla \text{C}{\left(\text{x}\right)}^{T}\alpha^{-1}\text{C}(\text{x}).$$

To solve the mass point position state at each timestep, we discretize equation (2.1) with timestep Δt, using *n* as an index of the current timestep:

2.4 $${\text{f}}_{\mathrm{constraint}}^{n+1}+{\text{f}}_{\mathrm{spring}}^{n+1}+{\text{f}}_{\mathrm{external}}^{n+1}=\text{M}\left(\frac{{\text{x}}^{n+1}-2{\text{x}}^{n}+{\text{x}}^{n-1}}{\Delta t^{2}}\right).$$

Similar to extended position-based dynamics (XPBD) [20], we introduce a Lagrange multiplier to decompose the force into its directional and scalar components. Further, we use the time interval and the inverse stiffness of constraint together to obtain $\tilde{\alpha }=\alpha /\Delta t^{2}$ for simplifying the description

2.5 $$\lambda =-{\tilde{\alpha }}^{-1}\text{C}(\text{x}).$$

Substituting equations (2.5) and (2.3) into equation (2.4) and rearranging the result yields

2.6 $$\nabla \text{C}{\left({\text{x}}^{n+1}\right)}^{T}\lambda^{n+1}=\text{M}({\text{x}}^{n+1}-({\text{x}}^{n}+(({\text{x}}^{n}-{\text{x}}^{n-1})+{\text{f}}_{\mathrm{spring}}^{n+1}+{\text{f}}_{\mathrm{external}}^{n+1})).$$

The right-hand side of the equation, which represents the predicted position, can be simplified as ${\text{x}}^{*}=({\text{x}}^{n}+(({\text{x}}^{n}-{\text{x}}^{n-1})+{\text{f}}_{\mathrm{spring}}^{n+1}\Delta t^{2}+{\text{f}}_{\mathrm{external}}^{n+1}\Delta t^{2})$ and is calculated prior to the constraint projection step. To determine the mass point position at timestep n+1, we must solve the following constraint-function-based minimization problem.

2.7 $$\text{g}({\text{x}}^{n+1},\lambda^{n+1})=\nabla \text{C}{\left({\text{x}}^{n+1}\right)}^{T}\lambda^{n+1}-\text{M}({\text{x}}^{n+1}-{\text{x}}^{*})=0$$

and

2.8 $$\text{h}({\text{x}}^{n+1},\lambda^{n+1})=\lambda^{n+1}\tilde{\alpha }+\text{C}({\text{x}}^{n+1})=0.$$

Analysing the above equations, our goal is to determine λ and **x** for timestep n+1. The number of functions is equal to the number of variables, so there is a unique solution. Because the constraint function is abstracted on the physical deformation behaviour, it is often not the linear form of mass point position. There is no analytical solution for mass point position directly. To solve these nonlinear equations, we linearize them with the Newton–Raphson method and solve them iteratively. We set the iteration count to *i* and eliminate the n+1 timestep:

2.9 $$\text{g}({\text{x}}_{i+1},\lambda_{i+1})=\text{g}({\text{x}}_{i},\lambda_{i})+\frac{\partial \text{g}}{\partial \text{x}}\;*\;\Delta \text{x}+\frac{\partial \text{g}}{\partial \lambda }\;*\;\Delta \lambda =0$$

and

2.10 $$\text{h}({\text{x}}_{i+1},\lambda_{i+1})=\text{h}({\text{x}}_{i},\lambda_{i})+\frac{\partial \text{h}}{\partial \text{x}}\;*\;\Delta \text{x}+\frac{\partial \text{h}}{\partial \lambda }\;*\;\Delta \lambda =0.$$

Our goal is to obtain Δx and Δλ in every iteration, ultimately using these values to update $\lambda_{i+1}=\lambda_{i}+\Delta \lambda$ and ${\text{x}}_{i+1}={\text{x}}_{i}+\Delta \text{x}$. This system requires two approximations (as does XPBD) that do not significantly influence the final solution. First, consider that when iteration count i=0, $\text{g}({\text{x}}_{i},\lambda_{i})=0$, and for large iteration counts, $\text{g}({\text{x}}_{i},\lambda_{i})$ remains small, making $\text{g}({\text{x}}_{i},\lambda_{i})$ negligible. Second, it is expensive to determine the second-order partial derivative of the constraint function:

2.11 $$\frac{\partial \text{g}}{\partial \text{x}}=\frac{\partial (\nabla \text{C}{\left({\text{x}}_{i}\right)}^{T}\lambda_{i})}{\partial \text{x}}-\text{M},$$

whereas rejecting the first part of ∂g/∂x has little influence on the convergence speed of the result. Therefore, it is reasonable to let ∂g/∂x=−M. For more detail, see [20]. Next, we can solve the linearized equations (2.9) and (2.10):

2.12 $$-\text{M}\Delta \text{x}+\nabla \text{C}{\left({\text{x}}_{i}\right)}^{T}\Delta \lambda =0$$

and

2.13 $$\lambda_{i}\tilde{\alpha }+\text{C}({\text{x}}_{i})+\nabla \text{C}({\text{x}}_{i})\Delta \text{x}+\tilde{\alpha }\Delta \lambda =0.$$

Then, we get $\Delta \text{x}={\text{M}}^{-1}\nabla \text{C}{\left({\text{x}}_{i}\right)}^{T}\Delta \lambda$ from equation (2.12), and substituting it into equation (2.13), get

2.14 $$\Delta \lambda =\frac{\lambda_{i}\tilde{\alpha }+\text{C}({\text{x}}_{i})}{\nabla \text{C}({\text{x}}_{i}){\text{M}}^{-1}\nabla \text{C}{\left({\text{x}}_{i}\right)}^{T}+\tilde{\alpha }}.$$

Once Δλ is solved, Δx can be determined easily.

Next, we integrate the MSD $f_{\mathrm{spring}}$, to model the nonlinearity and viscoelasticity of soft tissue. The nonlinear spring stiffness and mass point damper are introduced as follows. Here, we calculate the spring force per mass point *i*,

2.15 $$f_{\mathrm{spring}}=f_{\mathrm{damper}}+\overset{k}{\underset{j=0}{\sum }}f_{ij}$$

and $f_{\mathrm{damper}}$ as

2.16 $$f_{\mathrm{damper}}=(b_{0}+b_{1}*||x_{i}-x_{i}^{0}||)\dot{x}.$$

This direct velocity damp force to the point mass mimics viscoelasticity [21]. The spring elastic force is defined as

2.17 $$\begin{array}{rl} f_{ij} & =k_{ij}\frac{X_{i}-X_{j}}{\left||X_{i}-X_{j}|\right|}, \end{array}$$

2.18 $$\begin{array}{rl} k_{ij} & =\left\{ \begin{array}{ll} k_{1}\Delta l_{ij}+k_{2}\Delta l_{ij}^{3} & \left|\Delta l_{ij}\leq \Delta l_{c}\right|\\ \left(A+B(\left|\Delta l_{ij}|-|\Delta l_{c}\right|))\text{sgn}(\Delta l_{ij}\right) & \left|\Delta l_{ij}>\Delta l_{c}\right| \end{array}\right. \end{array}$$

2.19 $$\begin{array}{rl} \;\text{and}\;A & =k_{1}\Delta l_{c}+k_{2}\Delta l_{c}^{3}\;B=k_{1}+3k_{2}\Delta l_{c}^{2}. \end{array}$$

*k_ij_* corresponds to $\Delta l_{ij}=\;||x_{i}-x_{j}||-l_{ij}^{0}$. Therefore, spring stiffness is a third-degree polynomial at low displacements, and linear at higher displacements.

The concept of our new method is to separate the behaviour model into two components: nonlinear viscoelastic MSD reflect the general physical characteristics of soft tissue; and XPBD handles complex effects, such as incompressibility and maintains the models' stability. Control parameters are listed in table 1 for clarification.

**Table 1.** Physical model control parameters and their corresponding descriptions.

**parameter**	**description**
α	constraint stiffness
$b_{0}$	direct velocity damp for stability
$b_{1}$	viscoelasticity
$k_{1}$	nonlinear spring stiffness
$k_{2}$	linear spring stiffness

### 2.2. The algorithm

Using the derivations above, we can summarize the flow of our algorithm as follows. First, nonlinearity and viscoelasticity are reflected in the spring force calculation step and the extended spring force is introduced as external force to estimate the mass position. Then, the constraint projection loop step begins to modify the position to satisfy the constraint-function equation. We use XPBD to keep the deformation effect from depending on the iterator count. We benefit from using the Gauss–Seidel method, which maintains the stability of the final mass point's position.


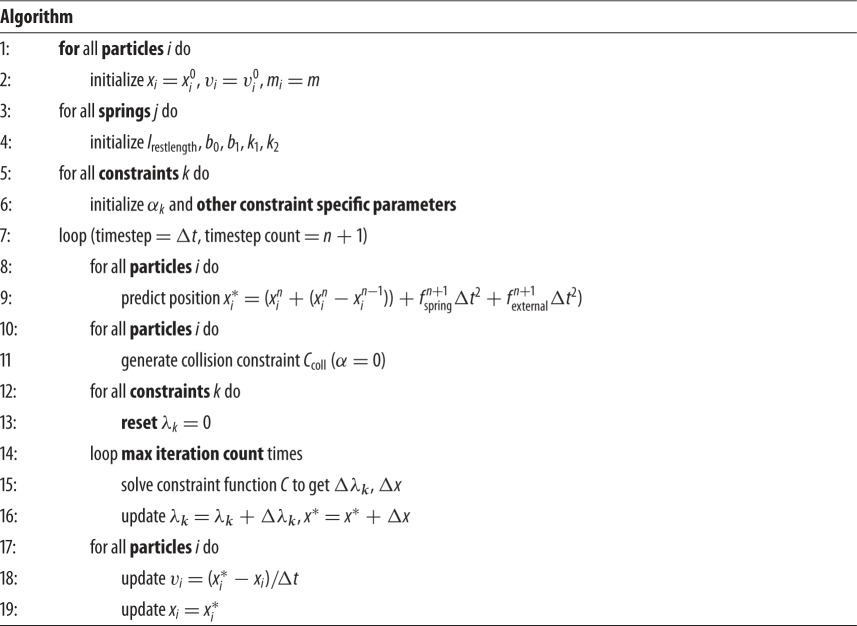


From the above steps, compared to conventional PBD, the only part that is added is the spring force calculated in the predicting position step. Thus, the time cost per timestep of this method is slightly higher than PBD. However, it is still acceptable and can meet the demand of real-time deformation applications, while achieving unconditional stability and modelling more complex deformation behaviours.

### 2.3. Collision detection and response

**Figure 2.**
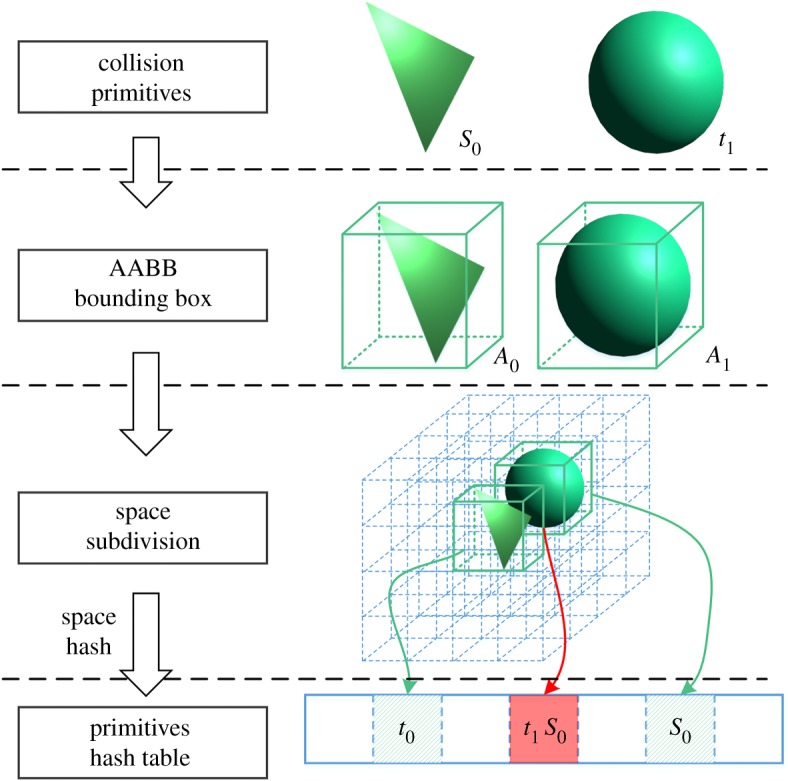
Detection of deformable body collision using the modified space hash algorithm.

A precise and fast collision detection method is crucial for avoiding penetration and triggering the deformation calculation, lest the trainee's immersion and concentration be disrupted. For this purpose, we use the modified space hash algorithm [22]. As shown in figure 2, in our model, the simulated objects are constructed as triangles and spheres.

First, we calculate the AABB bound box of all primitives. Second, the space hash algorithm is enforced to distribute the box index to a hash index array. Third, we traverse the hash array and execute collision detection for each element with the same index. The AABB bound box is only used to project primitive to the axial plane in the *x*-, *y*- and *z*-directions. We maintain a two-dimensional array for each primitive detection state to avoid repeat detection.

After collision detection is completed, collision response moves the primitives to a collision free state. In conventional PBD, two types of distance constraints are generated for collision response.

**Figure 3.**
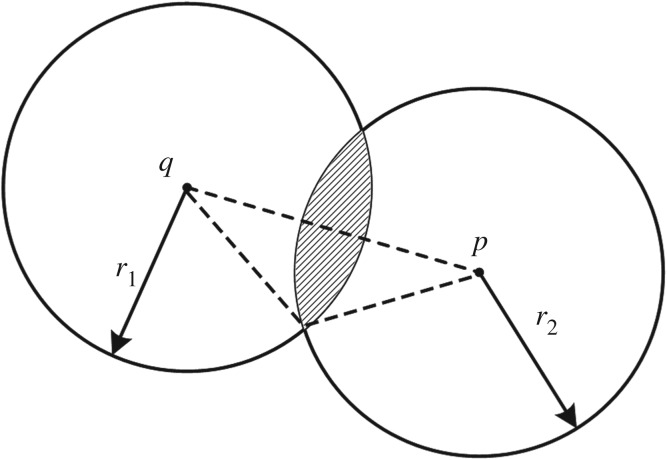
Collision detected between two spheres.

Sphere–sphere collisions, as in figure 3, can be handled as a vertex distance constraint. For sphere centre points *q* and *p*, and radii $r_{1}$ and $r_{2}$, the constraint function is

2.20 $$C(q,p)=|q-p|-r_{1}-r_{2}.$$

**Figure 4.**
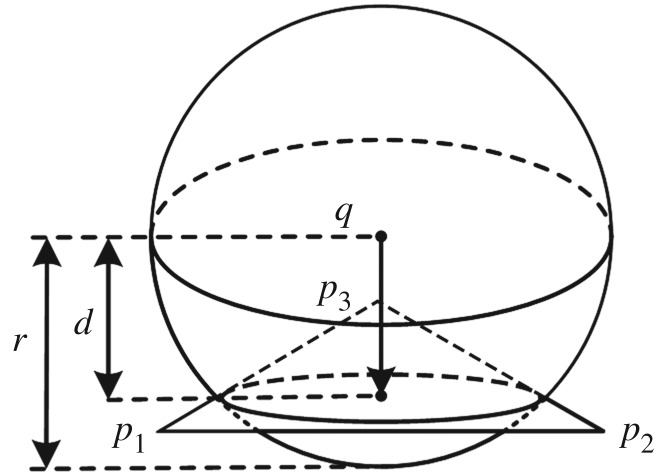
Collision detected between a sphere and triangle.

Sphere–triangle collisions and triangle–triangle collisions, demonstrated in figure 4, can be handled as a vertex and triangle distance constraint. The triangle–triangle collisions are resolved using three separate vertex and triangle distance constraints. For sphere centre point *q*; radius *r* and triangle points $q_{1}$, $q_{2}$ and $q_{3}$, the constraint function should be

2.21 $$C(q,p_{1},p_{2,}p_{3})=(q-p_{1})\cdot \frac{p_{2,1}\times p_{3,1}}{\left|p_{2,1}\times p_{3,1}\right|}-r.$$

After collision constraints are generated, they are added to later constraint projection steps for collision response calculation. For all collision constraint, α=0 to guarantee that the constraint equation is fulfilled as PBD.

### 2.4. Model example

There is a vital link between the structure of soft tissue and organ deformation behaviour. We can naturalistically simulate effects involving an organ's topological structure by introducing corresponding geometric constraints on mass point position. This technique enables our method to quickly and stably simulate complex behaviours. As an example, we demonstrate two typical organ models: the liver and gallbladder. The liver is an entity organ, meaning that it is composed of closely arranged cells. Conversely, the gallbladder is a cavity organ, composed of stratiform distribution surface cells. We use a closed triangular surface mesh to construct the topology of the gallbladder, and a tetrahedral volume mesh to construct the topology of the liver.

**Figure 5.**
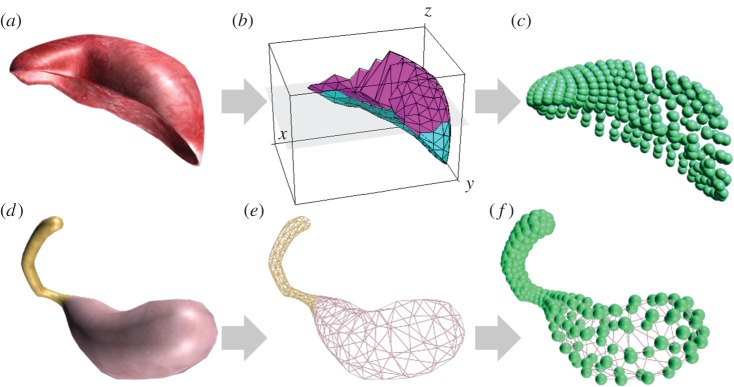
Generating physical models of the liver and gallbladder: (*a*–*c*) geometry data, tetrahedral mesh and physical data of the liver; (*d*–*f*) geometry data, surface mesh and physical data of the gallbladder.

In more detail, model expression is separating into three steps (figure 5). First, the organ surface mesh models are constructed from the ‘Virtual Chinese Human' colour slice dataset [23]. Second, for entity organs such as the liver, we generate a tetrahedral volume mesh. Finally, the basic elements for deformation calculating are generated from topology information such as nodes, springs and constraints. Different volume constraints are enforced for volume preservation when modelling the structural differences in the liver and gallbladder. Given the local volume preservation of liver, tetrahedral volume constraint is applied:

2.22 $$C(p_{1},p_{2},p_{3},p_{4})=\;\frac{1}{6}(p_{2,1}\times p_{3,1})\cdot \Delta p_{4,1}-V_{0}.$$

Similarly, to model the volume preservation of gallbladder, a closed surface mesh volume constraint is applied, as below:

2.23 $$C(p_{1},p_{2},\ldots ,p_{N})=\left(\overset{n_{\mathrm{triangles}}}{\underset{i=1}{\sum }}(p_{{t_{1}}^{i}}\times p_{{t_{2}}^{i}})\cdot p_{{t_{3}}^{i}}-V_{0}\right).$$

**Figure 6.**
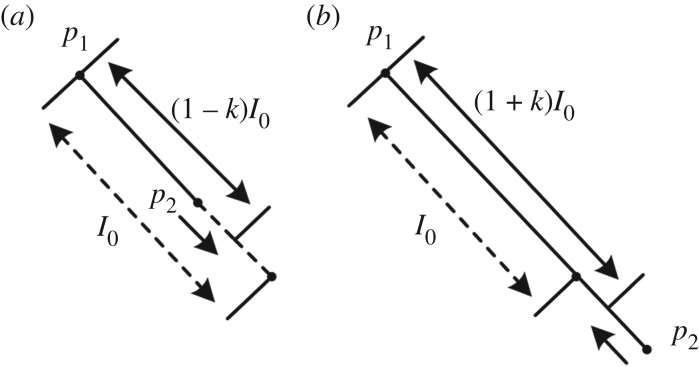
Overstretching constraint enforces *p*_1_ and *p*_2_ during compression (*a*) and stretching (*b*), respectively.

In the viscoelasticity calculation step of the mass spring model (MSM), there would be superelasticity effects on the spring that load a large force near the contact point (figure 6). To mitigate these effects, we introduce the below overstretching and compression constraints:

2.24 $$C(p_{1},p_{2})={\left(k_{\mathrm{ratio}}l_{0}\right)}^{2}-{\left(|p_{1}-p_{2}|-l_{0}\right)}^{2}.$$

By integrating the above constraints with the viscoelastic MSM, we can naturally simulate typical soft tissue deformation effects.

## 3. Results

### 3.1. Viscoelasticity and nonlinearity

**Figure 7.**
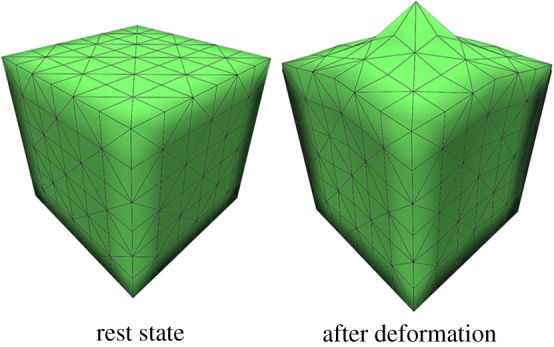
Cube model for testing viscoelasticity. The load point is at the centre of the upper-surface mesh.

Soft tissue, as a polymer, has nonlinear, creeping and stress-relaxing characteristics. To verify that the proposed integration model can represent these features, we built a simple cube model with 60 mm long edges for testing (figure 7). Each direction is composed of six cube primitives. The centre point of the upper surface was selected as a test point. The corresponding force load and displacement constraints are applied to it.

**Figure 8.**
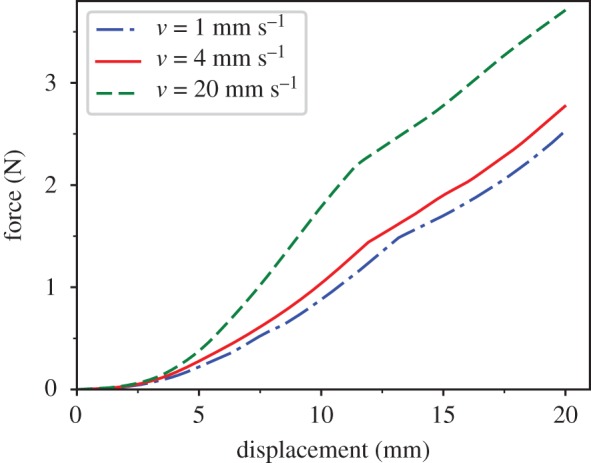
Force–displacement relationship reflecting nonlinear effects under a constant displacement velocity for parameter values *k*_1_ = 0.25, *k*_2_=10, *b*_0 _= 2 and *b*_1 _= 1000.

First, we tested the nonlinearity of the model. We applied a constant displacement velocity constraint to the test point. The direction of velocity was perpendicular to the upper surface. Then, through the force balance state of the test point, we obtain the force state. Figure 8 indicates that while the displacement is small, the relationship between displacement and force is nonlinear, and then it gradually transitions to a relatively linear relationship. When the displacement triggers the overstretching constraint, the slope of the figure is flat. Meanwhile, as the displacement velocity increases, the force increases. This phenomenon is consistent with the nonlinear characteristics of soft tissue.

**Figure 9.**
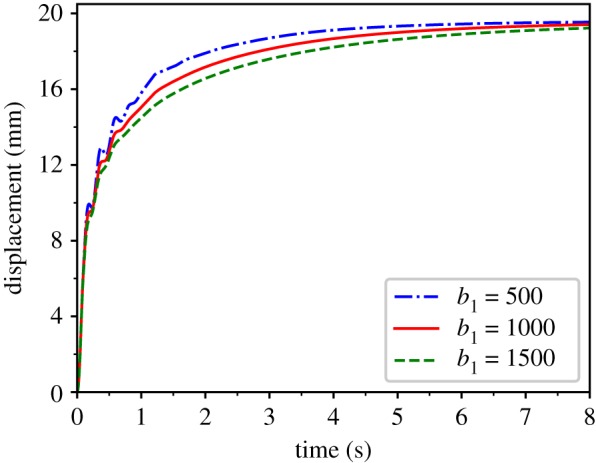
Displacement–time relationship reflecting creep under a constant external force for parameter values *k*_1 _= 0.05, *k*_2 _= 10 and *b*_0 _= 2.

Second, we tested creeping (figure 9). We still applied a constant force to the test point perpendicular to the upper surface. For the same values of *b*_1_, the displacement of the test point exhibits nonlinear growth over time. As *b*_1_ increases, the displacement of the test point is smaller, and finally, stabilizing to equal the displacement sample. This phenomenon is consistent with the creeping characteristics of soft tissue.

**Figure 10.**
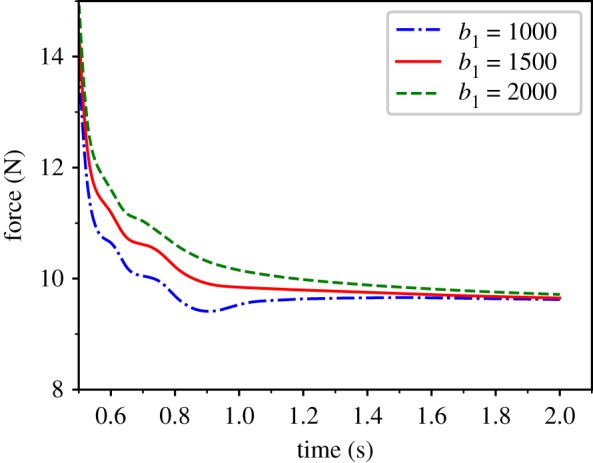
Force–time relationship reflecting stress-creep effects under a constant displacement for parameter values *k*_1 _= 1, *k*_2 _= 10, *b*_0 _= 2 and *α* = 0.

Third, we tested stress relaxation (figure 10). Before force sampling, the test point is moved to a same displacement of 20 mm over 0.5 s. Then, keeping the test point fixed, its force is sampled for 2 s. In the beginning of the test, due to the momentary displacement applied on test point, the instant elastic force loaded on the connected mass points around test point are much larger than the damping force, it causes the slight oscillatory response of the total loading force. But it quickly stabilized under the constraint of damping force by viscoelasticity. With the increase of the viscoelasticity coefficient *b*_1_, the trend of force declining becomes slower and the oscillatory response becomes weaker. The oscillatory response also can be optimized by variable timestep simulation. On the general trend, the model maintains the tendency of forcing decline over time, and the decay rate becomes slower as the viscoelasticity coefficient *b*_1_ increases. This phenomenon is consistent with the stress-relaxing characteristics of soft tissue.

It is not easy to find viscoelastic model parameter to directly correspond with the actual physical parameters. However, the effects of the model have the characteristic of real organs. Further, by adjusting the control parameters, the extent of the modelled organs' viscoelasticity changes consistently. Therefore, the proposing model meets the requirement of visual plausibility for visual surgery applications.

### 3.2. Comparative analysis of liver model

**Figure 11.**
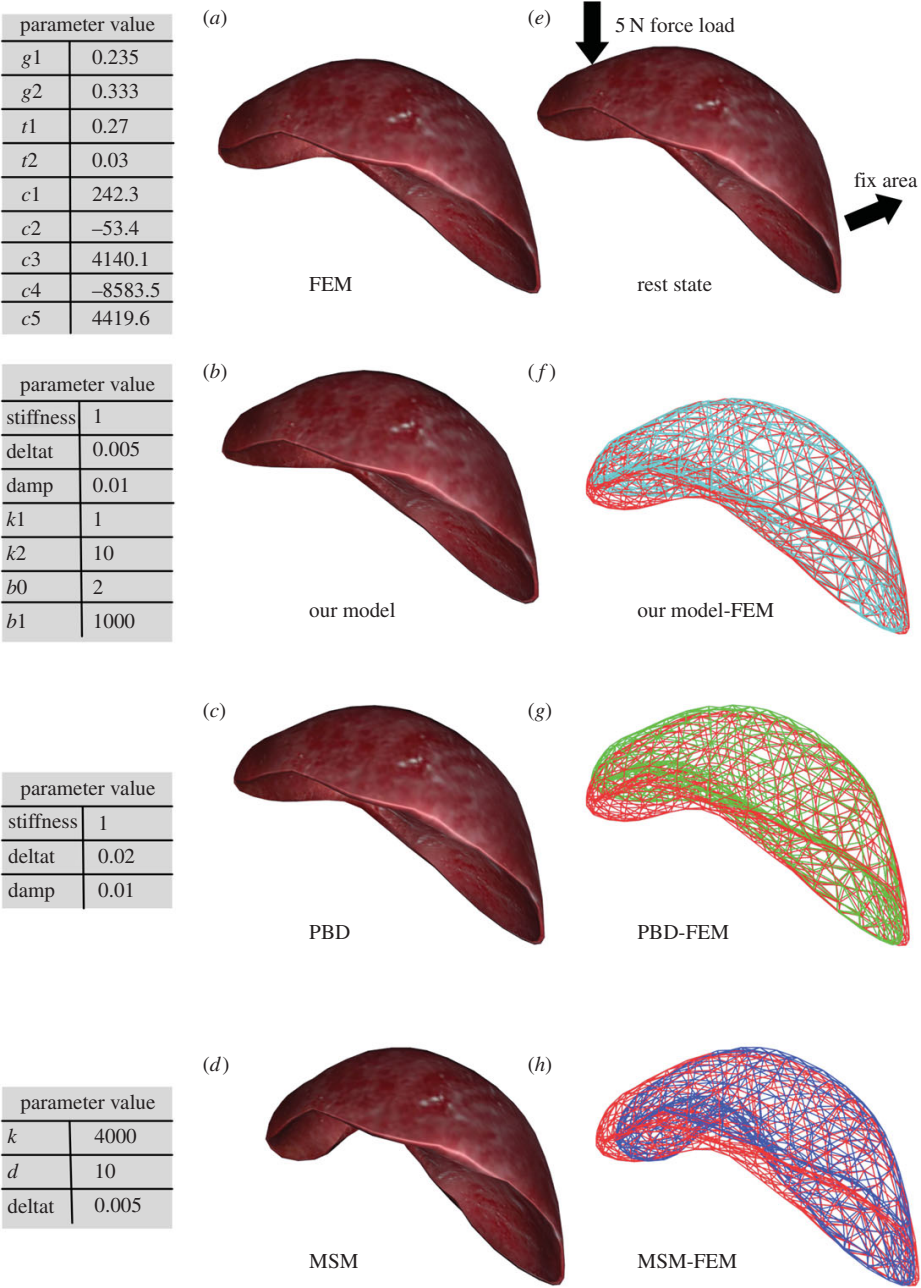
Comparison of the FEM model with our model, PBD and MSM. (*e*) The test case liver with fixed area and force load. (*a*–*d*) The stable state rendering picture of FEM, our model, PBD and MSM. (*f*–*h*) The surface wireframe of the corresponding models and the FEM model. The red colour wireframe represents the FEM model.

PBD and MSM are chosen to compare the numerical accuracy of our nonlinear viscoelastic model with that of the existing real-time model while ensuring visualization. To determine the simulation criteria, FEBio software is used to construct the constitutive model of the liver, which is based on the finite-element model (FEM). From [24,25], the Mooney–Rivlin model with viscoelasticity is used to represent the incompressible viscoelastic characteristics of the liver. The Mooney–Rivlin model is the finite-element model using polynomial forms of the strain energy. By extending the basic neo-Hookean model with the addition of the second invariant of the right Cauchy–Green strain tensors. It can be used to model material that exhibits some limited compressibility. The Mooney–Rivlin model with 2-Constant parameters models rubber well. The 5-Constant parameters of the Mooney–Rivlin model can be used to model liver. With the additional option of viscoelasticity, the soft tissue characteristics of liver can be reflected in a more comprehensive way. In table 2, the parameters of Mooney–Rivlin model of 5-Constant and viscoelastic model of 4-Constant are determined. During the test, the right part of the liver is enforced fixed constraints. The left part of liver is loaded by a 5 N force (figure 11*e*). Then, we record the final stable state of each model for comparison. From the rendered picture of the liver, our model is closest to FEM (figure 11*f*). The MSM model cannot ensure the incompressible which with large local deformation (figure 11*h*), and the deformation of PBD is relatively small owing to the strong constraint applied (figure 11*g*). In order to measure the global effect of each model, the whole surface node distance difference parameter *Q* is calculated, which is defined by the adding each node distance to the FEM standard.

3.1 $$Q=\overset{n}{\underset{i=0}{\sum }}|p_{\mathrm{model}}^{i}-p_{\mathrm{FEM}}^{i}|.$$

**Table 2.** The parameter description of finite-element model.

type	description	parameter name	value
**hyperelasticity**	5-Constant Mooney–Rivlin	*c*1	242.3
		*c*2	−53.4
		*c*3	4140.1
		*c*4	−8583.5
		*c*5	4419.6
**viscoelasticity**	relaxation times	*t*1	0.27
		*t*2	0.03
	viscoelastic coefficients	*g*1	0.235
		*g*2	0.333

Smaller *Q* demonstrates a smaller overall variance to the FEM model. It is found that the value of *Q* of our model is 2.57, that of PBD is 4.75 and that of MSM is 6.20. The above results show that our model has higher accuracy, while ensuring visual effects.

### 3.3. Volume preservation

**Figure 12.**
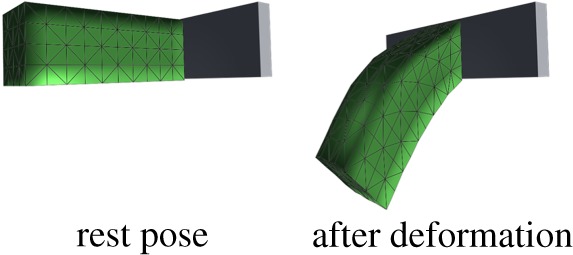
Cantilevered beam under gravity.

**Figure 13.**
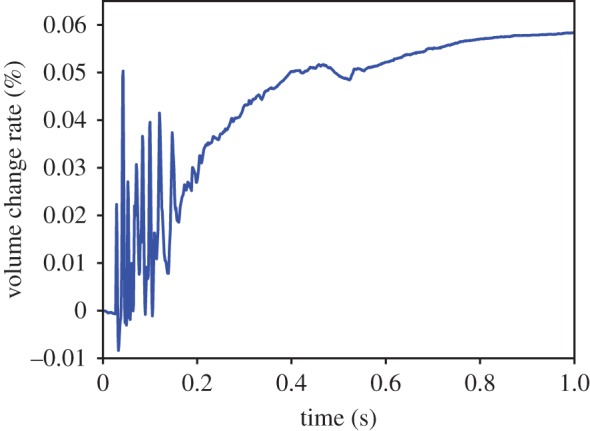
Volume change rate during cantilevered beam sagging.

**Figure 14.**
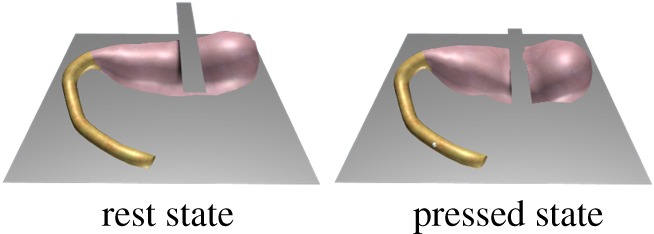
A gallbladder falls and is pressed by a plane.

**Figure 15.**
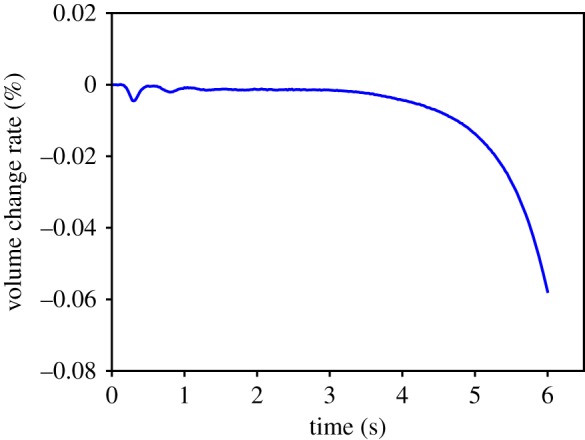
Volume change rate of a gallbladder as it falls and is compressed by a plane.

The structural features of the entity organs and cavity organs can be expressed by two different volume constraints. For the volume preservation effects of entity organs, we apply a local volume constraint. The tetrahedral volume constraint only influences the node position of related tetrahedra. Here, we use the cantilever beam model for testing by detecting the volume change of a cantilevered beam as it sags under gravity (figure 12). From the initial state (*t* = 0 s) to the natural sag (*t* = 1 s), the volume change ratio remained relatively low (figure 13). For volume preservation in cystic organs, we apply a global volume constraint. The closed surface volume constraint influences the position of all surface nodes at the same time. In this study, we use a falling gallbladder model for testing, by detecting the volume change of a gallbladder falling and pressed by a plane (figure 14). Owing to the enforcement of the global volume constraint, although the volume change ratio increases, it maintains an acceptable level (figure 15).

The two volume constraints above can constrain the volume of the model within acceptable levels, thus reflecting the structural features of the corresponding organ.

### 3.4. Stability

**Figure 16.**
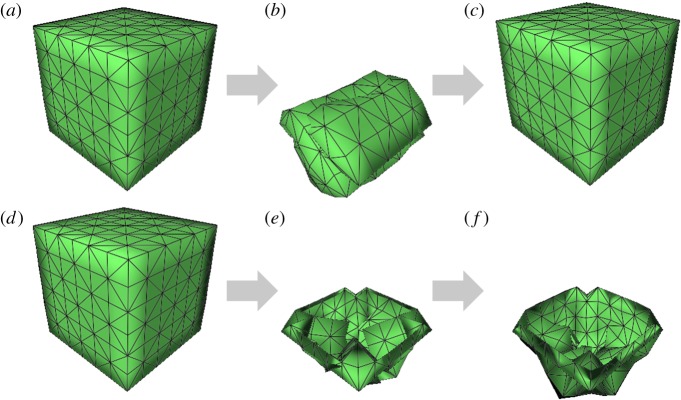
The cube model is compressed to an unstable state: (*a*–*c*) reflects that our model can recover its original shape; (*d*–*f*) reflects the inability of the conventional MSD to recover.

It is critical that soft tissue deformation methods for real-time virtual surgery applications can meet stability requirements. If unrealistic organ explosion occurs during interaction between the organs and instruments, the trainee's experience and immersion will be poor. Numerical integration errors make it easy to trigger such phenomena in traditional mass spring methods. By integrating PBD constraints into the spring force solution, we improve the stability of the model. In figure 16, we use an abnormal displacement constraint to produce an unrealistic model. Then, we release the constraint to obtain the recovery measure of the model. The result shows that our model is more stable than a conventional MSM.

### 3.5. Virtual laparoscopic cholecystectomy application

**Figure 17.**
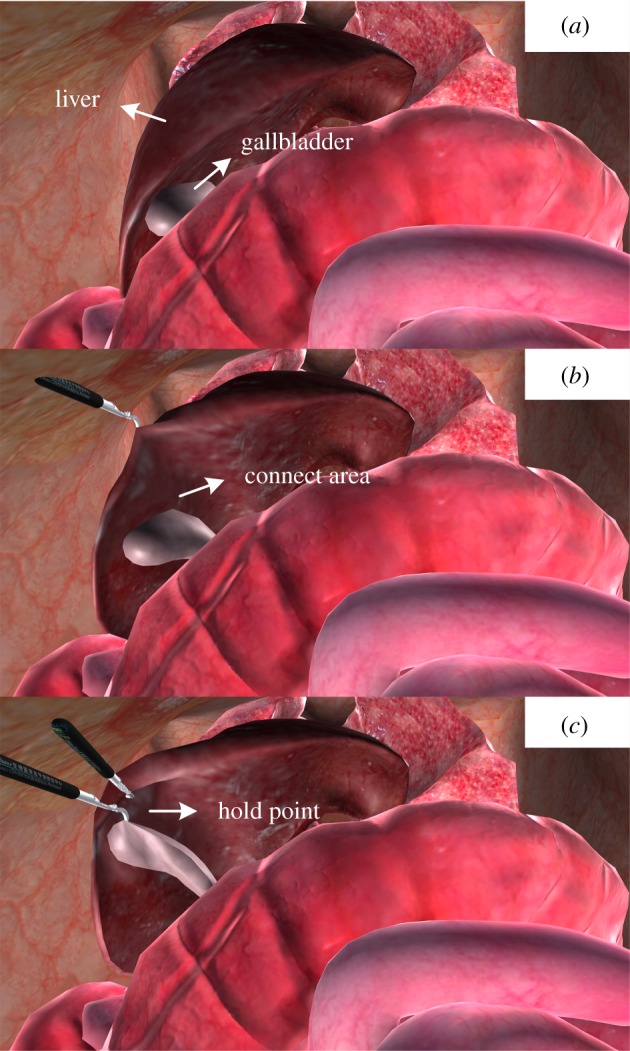
Laparoscopic scene views: (*a*) the resting state of the liver and gallbladder, (*b*) the gallbladder is dragged by its membrane with the liver using the clamp and (*c*) interactive operation clamping on the gallbladder.

**Figure 18.**
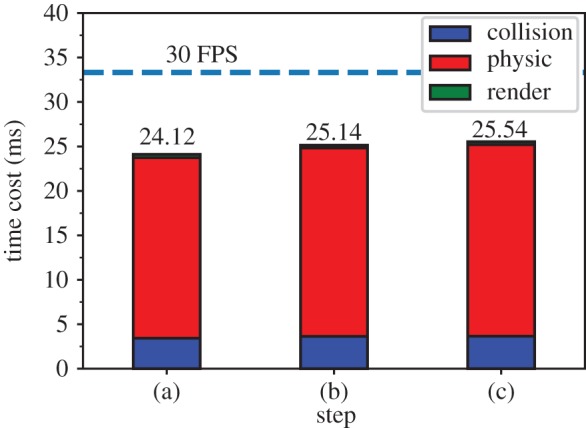
Average time cost per frame of corresponding steps in figure 17.

To verify the practicality of our method, we constructed a laparoscopic virtual surgery application. We built physical models for two important organs, the liver and gallbladder, using the proposed method. Viscoelastic springs, and local volume and overstretching constraints were used to model the entity organ (in this case, the liver). Viscoelastic springs, and global volume and overstretching constraints were used to model the cystic organ (the gallbladder). Our application was built on the Unity3D game engine [26]. The system runs on a Windows platform with Intel Core i7-7700 CPU @ 3.6 GHz and NVIDIA GTX 1070 GPU. The program runs in a single thread without multiple thread optimization for comparing computational costs. The key modules of the system include collision detection and deformation calculation, scene rendering and manipulator data acquisition and processing. The three steps in the cholecystectomy are shown in figure 17. Surgical scene information was recorded by the Unity3D frame rate profiler tool (table 3), indicating that a high frame rate (greater than 30 fps) was maintained during the operation. In figure 18, the average time cost per frame of three steps in figure 17 was recorded. From step (a) to step (b), and step (b) to step (c), the contacts between instruments and organs are increasing. Owing to collision response, the increase in constraint numbers leads to a slight increase in time cost. Nevertheless, the total time cost per frame is always below the real-time standard requirements (30 fps). Meanwhile, the fluctuation of time cost during the operation is small.

**Table 3.** Constructed data and time information captured from the Unity3D profiler.

item	category	value
**geometry data**	vertex	96.9 k units
	triangle	157.4 k units
**physical data**	node	650 units
	constraint	3163 units
	spring	1938 units
**collision primitive**	triangle	848 units
	sphere	248 units
**time cost**	render	0.3 ms
	physic	20.51 ms
	collision	3.45 ms

## 4. Conclusion

To simulate the viscoelasticity, nonlinearity and incompressibility of soft tissue deformation in real time, we have derived a new PBD method that integrates viscoelastic MSD. In this method, viscoelasticity and other time-related characteristics are simulated based on MSD. Then, the spring and external forces are combined with a constraint force generated by a PBD constraint function to correct the movement of particles. This method succeeds in controlling complex deformation behaviour through multiple constraint types, and is independent of iteration count and unconditionally stable. We generated a series of simple examples, which indicated our ability to control the extent of viscoelasticity through parameter adjustment. Finally, we applied this method to simulate soft tissue deformation in a laparoscopic cholecystectomy. This simulation showed the realistic deformation effects of the liver and gallbladder, and verified the practicality of the proposed method. The new method can meet the demand of modelling soft tissue deformation in real-time applications such as virtual surgery.

In future work, we plan to build a viscoelastic spring based on PBD through more controllable distance constraints. The XPBD framework has shown that the periodicity of a spring oscillator can be simulated by adjusting the parameters corresponding to the inverse stiffness of a realistic spring. Under extreme deformations, such as momentary displacement or force, the system has slight oscillations, which can be improved by introducing variable timestep simulation. Meanwhile, the variable timestep can be used for reducing time cost. Also, force feedback plays a significant role in measuring the effectiveness of the operation and enhancing the immersion of the trainers. Using the method proposed above, the feedback force in the deformation process can be easily solved by analysing the motion state of mass points. In the future application, we will add force feedback devices such as servo motors to produce reaction forces on the medical instrument and study the numerical accuracy of feedback force. Further, it would be worthwhile to build an overall relationship between model and physical parameters, as in the case of Young's modulus or Poisson's ratio.

## Data accessibility

FEBio software suite: (https://febio.org/) 3D Organ model data: liver and gallbladder (http://dx.doi.org/10.5061/dryad.1176k) [27].
